# Dracorhodin Perchlorate Regulates the Expression of Inflammatory Cytokines through the TLR4 Pathway and Improves Skin Wound Healing in Diabetic Rats

**DOI:** 10.1155/2022/9050686

**Published:** 2022-04-14

**Authors:** Zongliang Xiong, Mohan Huo, Yongzhen Jia, Chong Zhou, Xianglin Ma, Hang Yin, Xiaowen Jiang, Wenhui Yu

**Affiliations:** ^1^Department of Veterinary Medicine, Northeast Agricultural University, Harbin, Heilongjiang, China; ^2^Department of Life Sciences, Northeast Agricultural University, Harbin, Heilongjiang, China; ^3^Key Laboratory of the Provincial Education Department of Heilongjiang for Common Animal Disease Prevention and Treatment, Harbin, Heilongjiang, China; ^4^Institute of Traditional Chinese Veterinary Medicine, Northeast Agricultural University, Harbin, Heilongjiang, China

## Abstract

**Background:**

Dragon's blood is a natural medicine with hemostatic and blood-activating effects and is used to promote wound healing. Dracorhodin perchlorate (DP) is a stable form of dracarhod and is used as a substitute for cochinchinenin. DP promotes the proliferation of rat fibroblasts and promotes wound healing in rats.

**Methods:**

DP ointment (0.2 mg/mL) was applied to the skin wounds of nondiabetic and diabetic rats, and the skin of the wound was collected. Wound healing rate, H&E staining, Masson staining, TLR4 pathway, related inflammatory factors, nitric oxide synthase, and so forth were detected.

**Results:**

DP treatment alleviated the prolonged inflammatory cell infiltration time and the increase in the TLR4 pathway and inflammatory factors caused by diabetes. DP also promoted wound healing by increasing eNOS protein expression and NO content in the later stage of wound healing.

**Conclusion:**

DP promotes wound healing in diabetic rats by regulating the TLR4 pathway and related inflammatory factors. Therefore, adjuvant treatment of DP can be developed for diabetic wound healing.

## 1. Introduction

Diabetes can cause delayed wound healing for a variety of reasons. Hyperglycemia can lead to the overproduction of reactive oxygen species, leading to oxidative stress and mitochondrial dysfunction [1]. Hyperglycemia will affect the chemotaxis and phagocytosis of inflammatory cells, leading to the prolongation of the inflammatory process [2–4]. In addition, hyperglycemia can affect collagen deposition, decreased cell permeability, ischemia-reperfusion injury, decreased angiogenesis, and so forth [5, 6]. The process of wound healing is divided into three stages: inflammation, tissue proliferation, and new tissue remodeling [7]. In the early stages of healing, the inflammatory response favors wound decontamination, and macrophages, lymphocytes, and neutrophils are involved in this process [8, 9]. Macrophages phagocytose apoptotic neutrophils and secrete cytokines and growth factors that promote vascular regeneration, collagen deposition, and granulation. Cytokines such as TNF-*α*, IL-1*β*, and IL-6 are increased at the wound site [10], while cytokines such as IL-10 and IL-13 are decreased [11]. TLR4 is involved in LPS recognition and signal transduction and plays an important role in inflammatory immunity. TLR4 is involved in LPS recognition and signal transduction and plays an important role in inflammatory immunity. The TLR4 pathway regulates the expression of inflammatory factors such as IL-1*β*, TNF-*α*, COX-2, and iNOS [12]. IL-1*β* can promote fibroblast and endothelial cell proliferation, but its overexpression slows wound healing [13]. TNF-*α* is capable of chemotaxis of neutrophils, thermogenesis, and release of lysosomal enzymes, thereby causing tissue damage [14]. Increased COX-2 expression decreases collagen synthesis and delays the process of wound healing. Inhibition of iNOS and COX-2 expression suppresses the associated inflammatory response [15]. However, too strong or too long an inflammatory reaction will affect wound healing [8, 9, 16]. MyD88 is a major connector molecule in TLR signaling, and its death domain recruits downstream signaling molecules such as IRAK1, IRAK4, and TRAF6, which contribute to the activation of NF-*κ*B, AP-1, and P38 MAPK and subsequently induce transcription of proinflammatory cytokine-related genes [17]. TLR4 expression is increased in monocytes from diabetic patients and TLR-mediated inflammation is associated with glycated hemoglobin (HbA1c) [18].

NO (nitric oxide) is a lipid-soluble signaling molecule that is widely present in living organisms. It is transported without a transporter system and diffuses rapidly through the cell membrane to surrounding cells. In living organisms, NO is synthesized by three different isoforms of nitric oxide synthase (NOS) by catalyzing L-arginine [19]. Among the three NOS, endothelial NOS (eNOS) mainly regulates vascular tension [20]; neuronal NOS (nNOS) mainly transmits biological signals through synaptic structures [21]; inducible NOS (iNOS) is mainly involved in inflammatory responses and immune cell defense responses against pathogens [22]. All three NOS are expressed in skin tissue [23]. eNOS and iNOS are expressed in response to induction of cytokines, growth factors, and inflammation and are non-calcium-dependent [24–26]. iNOS can be continuously expressed in keratinocytes, Langerhans cells, fibroblasts, and endothelial cells through induction, especially after stimulation by cytokines or inflammatory factors, and induce a large amount of NO production to participate in the regulation of skin internal environment function, inflammatory response, and tissue damage repair [23, 27]. eNOS plays an important role in vascular regeneration after traumatic ischemia, mainly by promoting vascular smooth muscle proliferation and migration [28]. NO plays an important role in VEGF proliferation and migration of vascular endothelial cells, and NO is also involved in the provascular permeability of VEGF, and, conversely, NO can also increase VEGF synthesis by endothelial cells by increasing the activity of the VEGF gene promoter, which in turn promotes angiogenesis [29, 30].

Dragon's blood, a Chinese medicine, is made by processing resin exuded from the palm tree Kylin. The pharmacological effects of Dragon's blood mainly include hemostasis regulation, blood circulation promotion, and cardiovascular effects and are widely used clinically in blood activation and stasis resolution [31]. The research shows that the action direction and target of herbal drugs can be obtained by calculation methods and phytochemistry. This is worth learning from [32, 33]. Dracorhodin is the most important extract of Dragon′s blood and has a flavonoid structure. It is very unstable and easy to reduce and exists in the form of salt. Dracorhodin often exists in the form of Dracorhodin perchlorate (DP), where DP is widely used as a substitute to Dracorhodin in scientific research. Recent studies have shown that DP ointment can promote angiogenesis, increase the expression of EGF and VEGF proteins on the wound surface of rats, and regulate inflammation by reducing TNF-*α* [34]. In in vitro experiments, when siRNA interferes with ERK, DP cannot induce the proliferation of NIH-3T3 cells; DP can promote the proliferation of NIH-3T3 cells by regulating the ERK signaling pathway and thus promotes wound healing [35, 36].

In this experiment, we investigated DP intervention on wound healing in diabetic rats and examined the effect of DP on wound healing in diabetic rats by H&E staining, Masson staining, and microvessel formation. DP was examined to reduce persistent inflammation in diabetic wounds through the TLR4 pathway while increasing eNOS expression and NO content in the late wound stage.

## 2. Materials and Methods

### 2.1. Drug Preparation

Dracorhodin perchlorate (20 mg, Yuanye Bio, Shanghai, China) was dissolved in 2 mL of DMSO (Biotopped, Beijing, China), and 20 *μ*L of the mixture was dissolved in 980 *μ*L of DMSO. About 1 mL of the drug mixture was mixed with 16 g of Vaseline (Bodie Chemical Industry, Tianjin, China) to prepare ointment, which was stored at 4°C. DMSO ointment was also prepared by mixing 1 mL of DMSO and 16 g of Vaseline.

### 2.2. Animals

The experiment was designed by strictly following the guidelines of the International Laboratory Animal Care Evaluation and Appraisal Association. After obtaining the permission of the Research Animal Protection Committee of Northeast Agricultural University, 60 male SD rats (clean grade) weighing 170–220 g (Yisi Experimental Animal, Changchun, China) were raised under a 12 h/12 h light/dark cycle and given free diet and drinking water. The rats were divided into four groups: nondiabetic rat group (control group), diabetic rat group (STZ group), diabetic DMSO treatment group (STZ + DMSO group, 1 mL of DMSO mixed with 16 g of Vaseline), and diabetic DP treatment group (STZ + DP group, 200 *μ*g/mL). After 1 week of adaptive feeding, 45 of the rats were randomly selected and divided into three groups (*n* = 15). The rats were injected intraperitoneally with 60 mg/kg 1% STZ. After 7 days of injection, blood was taken from the rat tail tip to measure the blood glucose. Rats with blood glucose levels higher than 16.65 mmol/L were considered diabetic. Blood glucose was measured every 3 days during the wound healing test. The rats were anesthetized with 30 mg/kg Zoletil®50 (Virbac, Carros, France), and two full-thickness excision wounds were made on the back with a punch with a diameter of 1 cm. At 7, 14, and 21 days after the operation, five rats in each group were euthanized, and the skin of the wound area was taken for subsequent histological and biochemical analyses.

Wound healing was recorded on days 0, 3, 6, 9, 12, 15, 18, and 21. Wound healing rate (%) was calculated as (original wound area-wound area when photographed)/original wound area × 100.

### 2.3. Histological Analysis

The skin tissue was fixed in 4% paraformaldehyde and cut into coin-sized tissue slices, which were embedded in the tissues and made into pathological slices. The sections were deparaffinized and then subjected to hematoxylin-eosin staining and Masson staining. After mounting, the sections were magnified and photographed under an optical microscope (Nikon E100, Japan). Image-Pro plus 6.0 (Media Cybernetics, Inc, Rockville, MD, USA) was used to analyze wound healing, inflammation, and collagen deposition.

### 2.4. Immunohistochemistry

The paraffin sections were deparaffinized, antigen retrieval was performed, and endogenous peroxidase and serum were blocked for 30 min. The sections were added with 1 : 80 dilution of CD31 and COX-2 primary antibodies (Van Class, Shenyang, China) overnight at 4°C. After washing three times, the sections were incubated with secondary antibody (Bioss, Beijing, China) at room temperature for 50 min. After washing again, the sections showed color with DAB chromogenic solution. If the cells displayed brownish yellow color, then the result was positive. The nucleus was counterstained with hematoxylin and displayed blue. Finally, the sections were dehydrated, mounted, and examined under a microscope (Nikon E100, Japan). Image-Pro plus 6.0 (Media Cybernetics, Inc, Rockville, MD, USA) was used for immunohistochemical density analysis [37].

### 2.5. qRT-PCR

Rat skin tissue was placed in an electric homogenizer and ground. Total RNA was extracted with 1 mL of TRIzol (Wanlei, Shenyang, China), and cDNA was synthesized using HiScript III RT SuperMix for qPCR (Vazyme Biotech, Nanjing). qRT-PCR primers were designed based on the NCBI rat mRNA sequence database (Table 1). After the primers were synthesized, the RT-PCR reaction system was prepared according to the requirements of the Top Green qPCR SuperMix kit (TransGen Biotech, Beijing, China). The configured system was mixed and placed in a fluorescence quantitative PCR instrument (Roche, Shanghai, China) for testing [38].

### 2.6. Western Blot

The minced skin tissue, protein lysate (1 mL), and phenylmethylsulfonyl fluoride (PMSF; 10*μ*L) were placed in an electric homogenizer and ground to extract protein for measurement. The samples were stored at −80°C. After pretreatment, the protein was separated on 10% SDS-PAGE and transferred onto the PVDF membrane. The membrane was added with the primary antibody (Wanlei, Shenyang, China), incubated overnight at 4°C, and washed three times with TBST (TBS and 0.05% Tween). The membrane was then added with the secondary antibody (Boosen, Beijing, China) and incubated at 37°C for 2 h. The membrane was washed again with TBST three times and analyzed with an ECL chemiluminescence reagent. *β*-Actin (Wanlei, Shenyang, China) was used as a reference to analyze the protein expression levels of TLR4, MyD88, IRAK1, TRAF6, iNOS, and eNOS by using ImageJ software [39].

### 2.7. Skin Nitrite Content

According to the instructions of the nitric oxide determination kit (Jiancheng, Nanjing, China), 300 *μ*L of 10% homogenate supernatant was mixed with 200 *μ*L of reagent one and 100 *μ*L of reagent two. The mixture was mixed thoroughly, stood for 10 min, and centrifuged at 3500–4000 rpm for 15 min. The supernatant (160 *μ*L) was added with 80 *μ*L of the chromogenic agent, mixed well, and stood for 15 min. The OD values of each well were determined at 550 nm by a microplate reader (Gene, Hong Kong, China).

### 2.8. ELISA

According to the instruction manual of IL-1*β* and TNF-*α* ELISA kits (Lun Changshu, Xiamen, China), the tissue was mixed and ground with PBS to prepare 10% tissue homogenate. Standard wells were added with 50 *μ*L of different concentrations of the standard. Sample wells were added with 10 *μ*L of the test sample and 40 *μ*L of the sample diluent. Blank wells were added with 50 *μ*L of the sample diluent. The standard and sample wells were added with 100 *μ*L of horseradish peroxidase- (HRP-) labeled detection antibody. The reaction well was sealed by a film and incubated at 37°C for 60 min. The liquid in the wells was discarded. Each well was filled with the washing solution, which was then discarded. The reaction well was patted dry, and the process was repeated five times. All wells were added with 50 *μ*L of substrate A and 50 *μ*L of substrate B in turn and incubated at 37°C for 15 min in the dark. All wells were then added with 50 *μ*L of the termination solution. The OD values of each well were determined at 450 nm by a microplate reader (Gene, Hong Kong, China).

### 2.9. Data Analysis

GraphPad 8.0 was used for data analysis. The results were presented as mean ± standard deviation (mean ± SD). One-way ANOVA was used to test the difference between the data of each group through the least significant difference. In the figure and table, ^*∗*^ represents *p* < 0.05 compared with the control group at the same time point, and ^*∗∗*^represents *p* < 0.01 compared with the control group at the same time point. Moreover, # represents *p* < 0.05 between the STZ and STZ + DP groups at the same time point, and ## represents *p* < 0.01 compared with the STZ + DP group at the same time point.

## 3. Results

### 3.1. Clinical Observations

**Figure 1:**
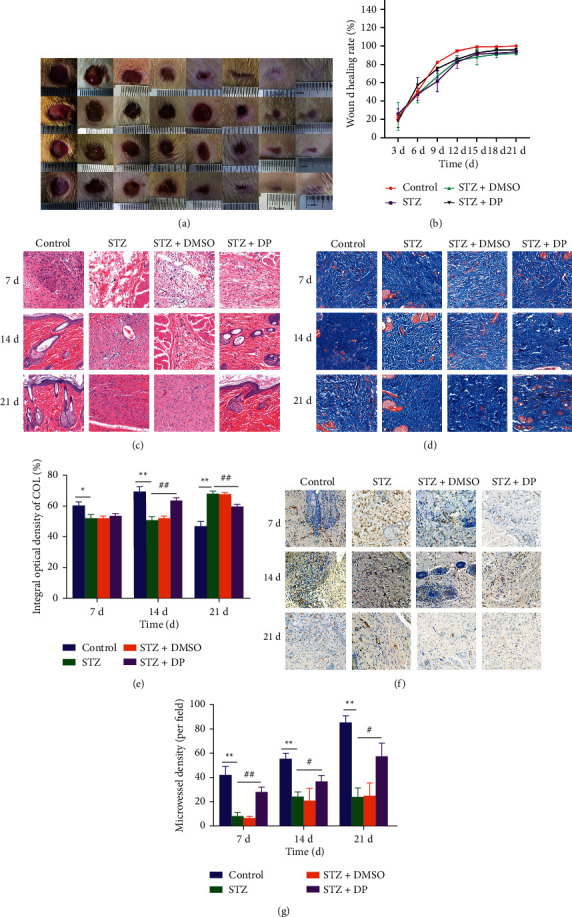
DP promotes skin wound healing in diabetic rats. (a) Representative wound photographs at different times after trauma; (b) wound healing rates at different times after trauma; (c) H&E staining of wound tissue at 7 d, 14 d and 21 d, 20x, 50 *μ*m; (d) Masson staining of wound tissue at 7 d, 14 d, and 21 d, 20x, 50 *μ*m; (e) collagen deposition rates of wound tissue at 7 d, 14 d, and 21 d; (f) CD31 positive staining of wound tissue at 7 d, 14 d, and 21 d; (g) 14 d and 21 d collagen deposition rate of wound tissue; (f) CD31 positive staining of wound tissue at 20x, 50 *μ*m, at 7 d, 14 d, and 21 d; (g) number of microvessels in wound tissue at 7 d, 14 d, and 21 d. *n* = 10; ^*∗*^*p* < 0.05, compared with control group. ^*∗∗*^*p* < 0.01, compared with the control group. Data are expressed as mean ± SEM.

The wound healing rate of STZ rats was overall lower than that of control rats, but the healing rate in the first 6 d was the same. The application of DP ointment accelerated the wound healing in STZ rats, which was higher than that of control rats in the first 7 d and lower than that of control rats in the last 14 d (Figures 1(a) and 1(b)). 21 d later, the wounds of control rats were all healed, while the wounds of STZ rats and STZ + DMSO rats were not healed, and only 2 rats in the STZ + DP group were left unhealed.

### 3.2. Histological Analysis of Wound Healing

Histological examination showed that, at 7 d, the control, STZ, STZ + DMSO, and STZ + DP groups all showed a small infiltration of lymphocytes and mast cells (black arrows), with thin epidermis and abundant dermal collagen. At 14 d, a small infiltration of inflammatory cells (black arrows) could still be seen in the STZ and STZ + DMSO groups, and vascular dilatation (yellow arrows) could be seen. The subcutaneous structures were clear in the control and DP groups, with hair follicles and sebaceous gland structures visible, and no obvious damage was seen. At 21 d, a small amount of inflammatory cell infiltration (black arrows) and a large number of new capillaries (red arrows) were still visible in the STZ and STZ + DMSO groups, and the wound was thicker than normal. The control and STZ + DP groups had thinner epidermis and no obvious damage was seen (Figure 1(c)).

### 3.3. Wound Healing Tissue Collagen Deposition

The type I collagen and type III collagen were stained blue by Masson staining, and the area of regional blue collagen fiber pixels and the area percentage of collagen fibers were measured and calculated using Image-Pro Plus 6.0 software. The results showed that the collagen deposition rate was greater in the control and STZ + DP groups than in the STZ and STZ + DMSO groups at the first 14 d. The collagen deposition rate was lower in the control and STZ + DP groups than in the STZ and STZ + DMSO groups at 21 d (*p* < 0.05), and more myofibrils could be observed in these two groups (Figures 1(d) and 1(e)).

### 3.4. Regeneration of Microvasculature in Wound Healing Tissue

The results of the microvessel density assay showed that the numbers of microvessels in the control and STZ + DP groups were significantly higher than those in the STZ and STZ + DMSO groups at 7 d, 14 d, and 21 d (*p* < 0.05), but the STZ + DP group could not recover to the level of the control group (Figures 1(f) and 1(g)). The difference between the STZ and STZ + DMSO groups was always insignificant, indicating that DMSO did not affect wound healing.

### 3.5. Expression of TLR4 Pathway-Related Proteins

**Figure 2:**
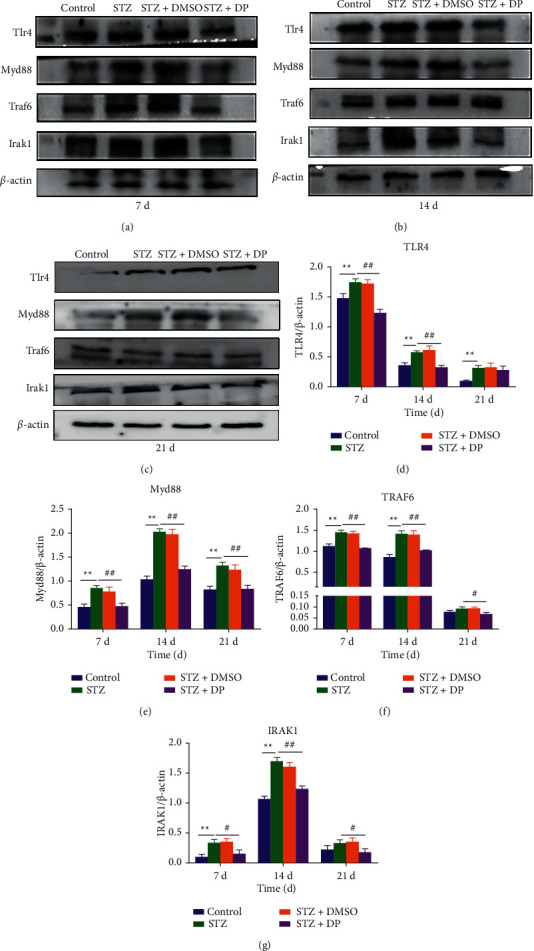
Effect of DP on protein expression of TLR4 pathway in rat skin. (a) Protein expression of TLR4 pathway in wound tissue at 7 d; (b) protein expression of TLR4 pathway in wound tissue at 14 d; (c) protein expression of TLR4 pathway in wound tissue at 21 d; (d) protein expression of TLR4 in wound tissue at 7 d, 14 d, and 21 d; (e) protein expression of Myd88 in wound tissue at 7 d, 14 d, and 21 d; (f) protein expression of TRAF6 in wound tissue at 7 d, 14 d, and 21 d; (g) protein expression of IRAK1 in wound tissue at 7 d, 14 d, and 21 d. *n* = 10; ^*∗*^*p* < 0.05 versus control group. ^*∗∗*^*p* < 0.01 versus control group. ^#^*p* < 0.05 versus STZ group. ^##^*p* < 0.05 versus STZ group. Data are expressed as mean ± SEM.

The TLR4 pathway regulates inflammatory factors such as IL-1*β*, TNF-*α*, and COX-2, so the protein expression of TLR4, Myd88, TRAF6, and IRAK1 was detected by protein immunoblotting, and the results showed that, at 7 and 14 d, compared with the control group, STZ treatment significantly elevated TLR4, Myd88, TRAF6, and IRAK1. The protein expressions of TLR4 and Myd88 were only increased at 21 d (*p* < 0.01). It indicates that hyperglycemia activated the TLR4 pathway during wound healing. In contrast, DP treatment significantly decreased the expressions of these proteins, except for TLR4, which was significantly reduced at all three time points (*p* < 0.05) (Figure 2). This indicates that DP reduced the activation of the TLR4 pathway in diabetic rat wounds.

### 3.6. Expression of Related Inflammatory Factors mRNA

**Figure 3:**
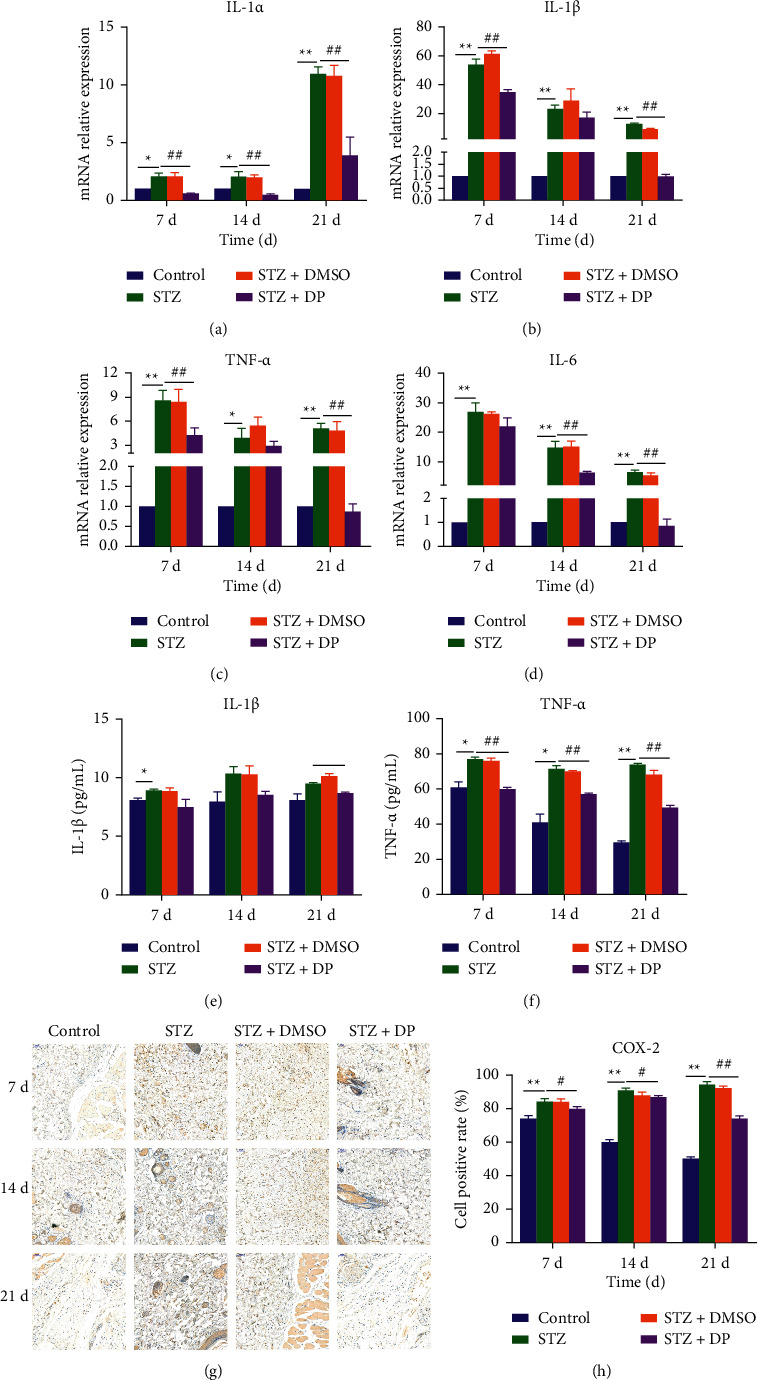
Effect of DP on inflammatory factors in rat skin. (a) mRNA expression of IL-1*α* in wound tissue at 7 d, 14 d, and 21 d; (b) mRNA expression of IL-1*β* in wound tissue at 7 d, 14 d, and 21 d; (c) mRNA expression of TNF-*α* in wound tissue at 7 d, 14 d, and 21 d; (d) mRNA expression of IL-6 in wound tissue at 7 d, 14 d, and 21 d; (e) protein content of IL-1*β* in wound tissue at 7 d, 14 d, and 21 d; (f) mRNA expression of IL-6 in wound tissue at 7 d, 14 d, and 21 d; (e) protein content of IL-1*β* in wound tissue at 7 d, 14 d, and 21 d; (f) protein content of TNF-*α* in wound tissue at 7 d, 14 d, and 21 d; (g) COX-2 positive staining in wound tissue at 7 d, 14 d, and 21 d, 20x, 50 *μ*m; (h) COX-2 positive staining in wound tissue at 7 d, 14 d, and 21 d COX-2 positive cell rate in tissue at 7 d, 14 d, and 21 d. *n* = 10; ^*∗*^*p* < 0.05 versus control group. ^*∗∗*^*p* < 0.01 versus control group. ^#^*p* < 0.05 versus STZ group. ^##^*p* < 0.05 versus STZ group. Data are expressed as mean ± SEM.

IL-1*α*, IL-1*β*, IL-6, and TNF-*α* have important regulatory roles in skin wound healing. Therefore, their mRNA expressions were measured using q RT-PCR. The results showed that the transcript levels of IL-1*α*, IL-1*β*, TNF-*α*, and IL-6 were significantly higher in the STZ group at 7 d, 14 d, and 21 d compared with the control group (*p* < 0.05) (Figures 3(a)–3(d)). mRNA expressions of inflammatory factors were significantly lower in the STZ + DP group compared with the STZ group (*p* < 0.05) and approached those of the control group at 21 d. IL-1*β*, TNF-*α*, and COX-2 are all downstream genes of the TLR 4 pathway, so their protein expressions were examined. ELISA results showed that DP reduced IL-1*β* levels in diabetic rat wounds mainly at 21 d (*p* < 0.05) and reduced TNF-*α* levels at all three time points (*p* < 0.01) (Figures 3(e)–3(f)). COX-2 content in wound skin was quantified by immunohistochemistry and it was demonstrated that DP decreased COX-2 expression at all three time points (*p* < 0.05), but it was still higher than the control group (Figures 3(g)–3(h)). Our study showed that DP decreased mRNA and protein expressions of inflammatory factors in the wound skin.

### 3.7. Expressions of NOS and NO in Skin Tissues

**Figure 4:**
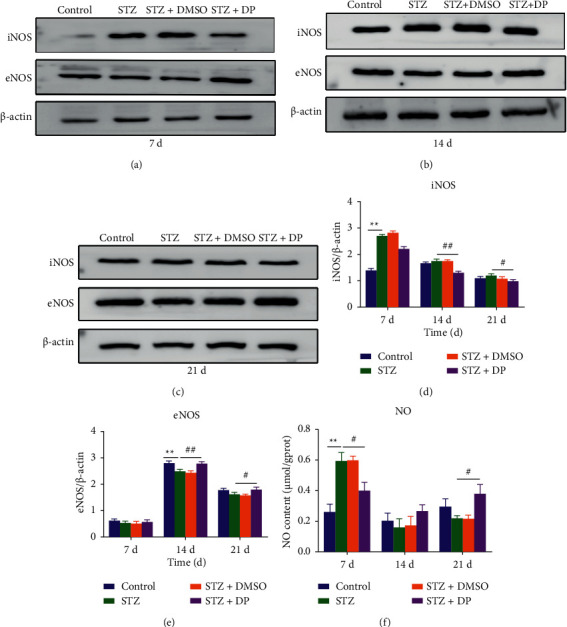
Effect of DP on NOS and NO expressions in rat skin. (a) Protein expression of iNOS and eNOS in wound tissue at 7 d; (b) protein expression of iNOS and eNOS in wound tissue at 14 d; (c) protein expression of iNOS and eNOS in wound tissue at 21 d; (d) protein expression of iNOS in wound tissue at 7 d, 14 d, and 21 d; (e) protein expression of eNOS in wound tissue at 7 d, 14 d, and 21 d; (f) NO content in wound tissue at 7 d, 14 d, and 21 d. *n* = 10; ^*∗∗*^*p* < 0.05 versus control group. ^*∗∗*^*p* < 0.05 versus control group. ^#^*p* < 0.05 versus STZ group. ^##^*p* < 0.05 versus STZ group. Data are expressed as mean ± SEM.

We detected the protein expressions of iNOS and eNOS by protein immunoblotting (Figures 4(a)–4(c)) and measured the NO content with a nitric oxide assay kit. The results showed that hyperglycemia increased the protein expression of iNOS in rat skin at 7 d. DP treatment significantly decreased the protein expression of iNOS at 14 d and 21 d (*p* < 0.05) (Figure 4(d)) and significantly elevated the protein expression of eNOS (Figure 4(e)). The NO content of the STZ group at 7 d was significantly higher than that of the Control group, while the NO content of the STZ + DP group was significantly at 14 d; the NO content of the groups was opposite to that at 7 d but not significantly different from that of the STZ group (*p* < 0.01). At 21 d, the DP treatment significantly increased the NO content (*p* < 0.05) (Figure 4(f)).

## 4. Discussion

In this study, DP can promote diabetic wound healing. This study is the first to investigate the effect of DP on diabetic wounds. First, the wound size of each group of rats was photographed and calculated at different time points. H&E staining and Masson staining were performed. Microvessel density was measured by immunohistochemistry. The results proved that the healing of rat skin wounds was delayed by hyperglycemia but improved by DP. The wound healing rate in the DP group is higher than that in the control group on the first 6 days. On the first 7 days, inflammatory cytokine infiltration was observed in each group. The infiltration of inflammatory cytokines was detected in the STZ and STZ + DMSO groups throughout the wound healing process. Meanwhile, no inflammatory cytokines were observed in the control and DP treatment groups on 14 days, and complete hair follicles and sebaceous gland structures were observed. This finding proves that DP can improve skin wound healing by inhibiting inflammation. One of the important reasons for the difficulty of wound healing in diabetic patients is the infiltration of a large number of neutrophils and the intensified inflammation state [40]. Neutrophils can secrete a variety of proteolytic enzymes, such as serine proteinase, matrix metalloproteinase-2 (MMP-2), and matrix metalloproteinase-9 (MMP-9), to clean wounds. These proteolytic enzymes continue to degrade the proteins in the extracellular matrix (ECM), making it difficult for wounds to heal [41]. The Masson staining results showed that the collagen content in the control and DP treatment groups is higher than that in the STZ and STZ + DMSO groups on the first 14 days and is lower on 21 days. The deposition of collagen is one of the important processes of tissue repair, and collagen is one of the key components of ECM. The excessive deposition of collagen can also cause granulation tissue hyperplasia and scar formation [42]. On the first 14th day, the rate of collagen deposition in the DP treatment group is significantly higher than that in the STZ group, and collagen filled the wound area. On 21 d, the collagen content in the STZ and STZ + DMSO groups is higher than that in the control and DP treatment groups. This finding proves that diabetic wounds tended to form scars, and DP treatment inhibited the formation of wound scars. The microvessel density results also confirmed that DP accelerated the regeneration of new blood vessels, delivered nutrients to the wound, and accelerated wound healing [43]. DP can promote angiogenesis by increasing the expression of N, which can stimulate endothelial cells and macrophages to secrete vascular endothelial growth factor (VEGF) and basic fibroblast growth factor (bFGF) [44]. bFGF and VEGF are considered important mediators of angiogenesis and lymphangiogenesis [45]. In addition, DP can upregulate the expression of the VEGF protein in rat skin [34].

In the results of H&E staining, the DP group has less inflammatory cytokine infiltration than the STZ group. The expressions of the TLR4 pathway and inflammatory factors in rat skin were further determined to study whether DP accelerates wound healing in diabetic rats through anti-inflammatory methods. Diabetes increased TLR4-mediated inflammation, whereas DP inhibited the protein expression of the TLR4 pathway (TLR4, Myd88, IRAK1, and TRAF6) and reduced the mRNA expressions of IL-1*β*, IL-1*α*, IL-6, and TNF-*α* and the protein expressions of TNF-*α*, IL-1*β*, and COX-2. iNOS improves the inflammatory level of skin wounds. The TLR4 pathway plays an important role in skin wound healing. Defects in TLR4 and MyD88 and bone marrow-specific TLR4 defects can lead to poor wound healing and reduced expression of inflammatory cytokines required for wound repair [17]. However, in diabetic animals, hyperglycemia and ischemia lead to a significant increase in the expression and activation of TLR4, thereby promoting inflammation through downstream cytokines (such as IL-6) [46]. Reducing TLR4 in diabetic mice can reduce inflammation and improve wound healing, indicating that sustained TLR4 activation may be detrimental to diabetic wounds [47]. The TLR4 pathway regulates the expression of inflammatory cytokines, such as IL-1*β*, TNF-*α*, COX-2, and iNOS [12]. IL-1 can promote the proliferation of fibroblasts and endothelial cells, but its excessive expression will slow down wound healing [13]. TNF-*α* can chemoattract neutrophils, cause heat, and promote the release of lysosomal enzymes to cause tissue damage [14]. Increased COX-2 expression will reduce collagen synthesis and delay wound healing [7]. Suppressing the expressions of iNOS and COX-2 can inhibit inflammation [15]. In the present work, DP inhibited the expressions of IL-1*β*, TNF-*α*, iNOS, and COX-2 and reduced wound inflammation. Other experiments have shown that DP can reduce the expressions of IL-1*α* and TNF-*α* in normal rats in the early stage of wound healing [34]. In short, persistent inflammation in diabetic wounds hinders the wound from the inflammatory phase to the proliferation phase. The inhibition of the TLR4 pathway and related inflammatory cytokines provide new therapeutic targets for the treatment of diabetic wound healing.

iNOS is an enzyme that catalyzes the production of NO from L-arginine. NO content in the rat skin was detected. Interestingly, the NO content in the STZ + DP group is higher than that in the STZ group on day 21. In addition, the protein expression of eNOS is opposite to that of iNOS. This finding proves that the source of NO in the control and DP groups is eNOS. iNOS is only produced after acute inflammation stimulation [48]. NO concentration produced by iNOS corresponds to a proinflammatory effect, and NO concentration produced by eNOS corresponds to the anti-inflammatory process and plays an important role in the proliferation and maturation phases [49, 50]. NO has a vasodilator effect and can promote angiogenesis as well as the migration and proliferation of fibroblasts [51] and epithelial cells [52]. In the later stages of inflammation, NO significantly inhibits the expression of chemokines (RANTES) and human monocyte chemotactic protein-1 (MCP-1), which mediate the expression and secretion of activated normal T cells [53]. Experiments have shown that the expression of eNOS and the availability of NO in diabetic rats are lower than those in normal rats and affect skin wound healing. The increased expressions of eNOS and NO can inhibit wound O_2_- levels and improve wound healing [54]. This test proves that DP enhanced the expression of eNOS in diabetic wounds and increased the synthesis and utilization of NO in the late stage of wound healing. This test only measures the mechanism of DP in accelerating the healing of diabetic wounds from the aspect of inflammation. The effect of DP on the expression of growth factors should be further studied. In addition, diabetes can cause increased oxidative stress on skin wounds. In the future, the effect and mechanism of DP on reducing oxidative stress should be investigated to develop effective treatment strategies for diabetic wounds.

## 5. Conclusion

This study first verified the effect of DP on the skin wounds of diabetic rats. DP can promote skin wound healing, collagen deposition, and microvascular formation in diabetic rats. DP can reduce inflammation in wound tissues by inhibiting the TLR4 pathway and related inflammatory factors. DP can increase eNOS protein expression and NO content and improve the low utilization rate of NO caused by diabetes.

## Figures and Tables

**Table 1:** The sequences of primers used for quantitative real-time PCR.

Gene	Primer (5′⟶3′)
TNF-*α*	Forward: TGGGCTCCCTCTCATCAGTTCC
	Reverse: GCTCCTCCGCTTGGTGGTTTG
IL-6	Forward: GAGACTTCCAGCCAGTTGCC
	Reverse: ACTGGTCTGTTGTGGGTGGTA
IL-1*β*	Forward: ACAGCAGCATCTCGACAAGAGC
	Reverse: CCACGGGCAAGACATAGGTAGC
IL-1*α*	Forward: GGAGAGCCGGGTGGTGGTG
	Reverse: GGTGCTGATCTGGGTTGGATGG

## Data Availability

Some or all data, models, or codes generated or used during the study are available from the corresponding author by request.

## Abbreviations

DP: Dracorhodin perchlorate
ECM: Extracellular matrix
DMSO: Dimethyl sulfoxide
PVDF: Polyvinylidene fluoride
IL-1*α*: Interleukin-1*α*
IL-1*β*: Interleukin-1*β*
IL-6: Interleukin-6
TNF-*α*: Tumor necrosis factor-*α*
COX-2: Cyclooxygenase-2
ELISA: Enzyme-linked immunosorbent assay.
